# Characterisation and Harnessing of 5‐Hydroxymethylfurfural Metabolism in *Pseudomonas umsongensis* GO16 for the Production of 2,5‐Furandicarboxylic Acid

**DOI:** 10.1111/1751-7915.70159

**Published:** 2025-05-10

**Authors:** Rhys Orimaco, Pauric Donnelly, Seán Sexton, Aoife McLoughlin, Sophie Kelly, Kevin E. O'Connor, Nick Wierckx, Tanja Narančić

**Affiliations:** ^1^ UCD Earth Institute and School of Biomolecular and Biomedical Science University College Dublin Dublin 4 Ireland; ^2^ BiOrbic ‐ Bioeconomy Research Centre Ireland, University College Dublin Dublin 4 Ireland; ^3^ Institute of bio‐ and Geosciences IBG‐1: Biotechnology Forschungszentrum Jülich Jülich Germany

**Keywords:** 2,5‐Furandicaboxylic acid (FDCA), 5‐Hydroxymethylfurfural (HMF), biotransformation, plastics, polyethylene terephthalate (PET), *pseudomonas*, upcycling

## Abstract

In the search for biobased alternatives to traditional fossil plastics, 2,5‐furandicarboxylic acid (FDCA) represents a potential substitute to terephthalic acid (TPA), a monomer of the ubiquitous polyester, polyethylene terephthalate (PET). *Pseudomonas umsongensis* GO16, which can metabolise TPA and ethylene glycol (EG), can also oxidise 5‐hydroxymethylfurfural (HMF), a precursor to FDCA. The enzymes involved in the oxidation to FDCA, PsfA and PsfG, were identified and characterised. Deletion of FDCA decarboxylase HmfF involved in the conversion of FDCA to furoic acid, and subsequently to a central metabolic intermediate, 2‐ketoglutarate, allowed for the accumulation of FDCA. GO16 Δ*hmfF* cells were grown on glycerol, TPA, EG or mock PET hydrolysate, and the catalyst was then used for the biotransformation of HMF to FDCA. When TPA was used as a growth substrate and to power the biotransformation, the transport of 5‐hydroxymethyl‐2‐furancarboxylic acid (HMFCA) into the cytoplasm represented a rate‐limiting step in HMF oxidation. De‐bottlenecking transport limitations through *in trans* overexpression of the HMFCA transporter (HmfT) along with the PsfA aldehyde dehydrogenase and PsfG alcohol dehydrogenase allowed 100% conversion of 50 mM HMF to FDCA within 24 h when TPA, EG or mock PET hydrolysate were used to grow the biocatalyst and subsequently to power the biotransformation. This expands the repertoire of valuable products obtained from engineered *P. umsongensis* GO16 in the strategy to bio‐upcycle post‐consumer PET.

## Graphical abstract

A new route for PET upcycling to 2,5‐furandicarboxylic acid (FDCA) was developed using an engineered *Pseudomonas umsongensis* GO16. This work will expand the scope of biotechnological upcycling of waste residues, contributing to the sustainable vision of the circular bioeconomy.

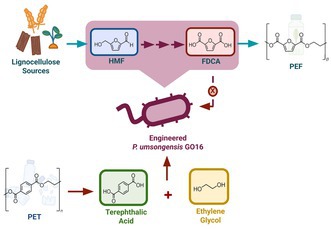

## 1 Introduction

Polyethylene 2,5‐furanoate (PEF) represents an alternative to the omnipresent plastic polyethylene terephthalate (PET). Both PEF and PET maintain similar physical and thermoplastic characteristics, such as high gas barrier properties which make them particularly suited for food and beverage packing applications (Loos et al. 2020; De Jong et al. 2022). The two molecules differ in their monomeric composition: while both contain ethylene glycol (EG), PEF contains 2,5‐furandicarboxylic acid (FDCA) as opposed to terephthalic acid (TPA) in PET. Whereas TPA is derived from petroleum‐based sources, FDCA can be produced from molecules derived from lignocellulose such as the biobased ‘sleeping giant’ 5‐hydroxymethylfurfural (HMF) (Werpy et al. 2004). Using FDCA instead of TPA thus offers a biobased alternative to PET while simultaneously improving the mechanical recycling potential of the resulting polymer (Sousa et al. 2015; Eerhart et al. 2012). The major hindrance to PEF's widespread adoption is its price: FDCA is expensive to produce using current thermocatalytic methods which rely on noble metal catalysts (Hwang et al. 2020). While the major production cost of 54% is contributed to HMF production, where feedstocks and utility requirements play a key role (Davidson et al. 2021), the cost of the conversion of HMF to FDCA is also a significant contributor. In the lignocellulosic biomass conversion into HMF, and then FDCA, the second step cost was mainly attributed to Pt/ZrO_2_ catalyst (Triebl et al. 2013). The decrease in minimum selling price for FDCA could come from the use of biorefineries, where FDCA production could be coupled with other valuable products, such as levulinic acid, lactic acid, or even protein (Davidson et al. 2021). Further optimisation of the processes that include the reduced use of the catalysts, solvents and associated equipment, is seen as a means to further decrease the minimum selling price of FDCA.

**FIGURE 1:**
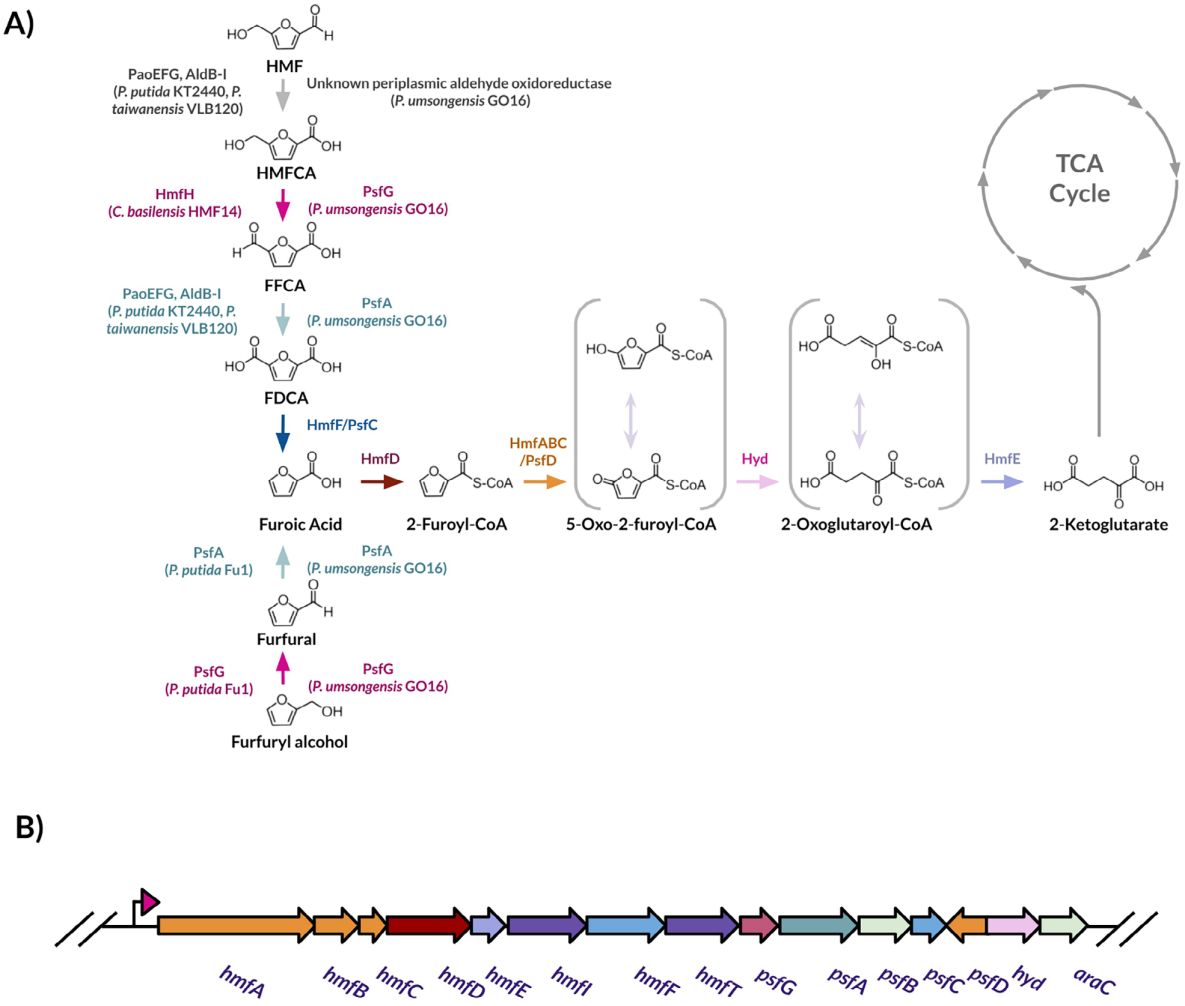
HMF metabolism in bacteria. (A) The HMF metabolic pathway, responsible for the catabolism of HMF and furfuryl alcohol into 2‐ketoglutarate via 2‐furoic acid. Enzymes specific to different strains with analogous roles are listed. (B) Genetic organisation of the *P. umsongensis* GO16 *hmf* cluster.

While a promising molecule in a biorefinery context, HMF's usage is hindered due to the toxicity conferred to it by the aldehyde functional group. As potent electrophiles, aldehydes can react with DNA or amino acids, forming adducts which can wreak havoc on cellular processes (Kunjapur and Prather 2015). In addition, attempts to detoxify these aldehydes may drain reducing power away from central metabolism, further crippling the cell (Miller et al. 2009). In spite of these effects, bacteria such as *Cupriavidus basilensis* HMF14, *Raoultella ornithilytica BF60* and *Pseudomonas putida* ALS1267 can efficiently metabolise HMF (Koopman et al. 2010b; Hossain Gazi et al. 2017; Crigler et al. 2020). In these organisms, HMF is sequentially oxidised to 5‐hydroxymethyl‐2‐furancarboxylic acid (HMFCA), formylfurancarboxylic acid (FFCA) and FDCA (Figure 1A). One of the carboxylic acid groups of FDCA is then cleaved, producing 2‐furoic acid, completing the upper pathway. The furan ring of 2‐furoic acid is then cleaved, generating the TCA cycle intermediate 2‐ketoglutarate in the lower pathway (Wierckx et al. 2011). Seminal work in the microbial conversion of HMF was carried out using *C. basilensis* HMF14. After annotating the *hmf* catabolising operon of this organism, heterologous expression of HMF oxidising enzymes and an associated transporter in the solvent tolerant strain *P. putida* S12 enabled fed‐batch production of FDCA (Koopman et al. 2010a). Other bacteria, such as *R. ornithilytica* BF60, *Escherichia coli* , *Gluconobacter oxydans* and *Mycobacterium* sp. MS1601 have since been used as chasses for the bioproduction of FDCA (Yuan et al. 2018; Kawanabe et al. 2021; Sayed et al. 2019). In each of the above cases, the organisms were grown on defined carbon sources such as glucose or glycerol which can be obtained from biomass or biodiesel manufacture. Nevertheless, expanding the range of microbial feedstocks to include waste residues could help lower the production costs of FDCA while also contributing to a circular economy (Tiso et al. 2022).

Despite plastics being relatively new to nature, several bacteria such as *Ideonella sakaiensis, Comamonas* sp. E6 and *Pseudomonas putida* KT2440 have already demonstrated the ability to grow on plastic monomers (Yoshida et al. 2016; Sasoh et al. 2006; Li et al. 2019). *P. umsongensis* GO16 is one such microbe that has the native capacity to consume both TPA and EG as carbon and energy substrates (Narancic et al. 2021). Discovered from soils surrounding a plastic bottling facility in Ireland, *P. umsongensis* GO16 grown on these molecules has previously been able to support the production of polyhydroxyalkanoates (Kenny et al. 2008; Kenny et al. 2012) and hydroxyalkanoyloxy‐alkanoates for the production of biobased polyurethane (Tiso et al. 2021). Building on these foundations, we seek to expand GO16's biocatalytic repertoire, through the generation of new biobased molecules that can be produced from the energy and biomass this organism accumulates from PET monomers. As well as TPA and EG, *P. umsongensis* GO16 has the capacity to catabolise a vast array of other molecules, including aromatics such as naphthalene, phenol and benzoic acid (Narancic et al. 2021). We have now identified and functionally characterised the HMF oxidation pathway of *P. umsongensis* GO16 in the context of FDCA production. PsfA, a putative aldehyde dehydrogenase, and PsfG, a putative alcohol dehydrogenase, are encoded in the *hmf* cluster (Figure 1B) and we have demonstrated their role in HMF oxidation to FDCA (Figure 1A). Further manipulation of the *hmf* cluster by deleting the FDCA decarboxylase encoding gene, *hmfF*, prevents through‐conversion of FDCA to 2‐furoic acid and further metabolism, allowing for FDCA accumulation. Finally, we benchmarked the glycerol powered biotransformation of HMF to FDCA by GO16 Δ*hmfF* against cells grown on TPA, EG and mock PET hydrolysate (corresponding to equal carbon amounts of both monomers). By generating the strain GO16 TGA, overexpressing key HMF upper pathway proteins, we significantly improved the conversion efficiency of HMF to FDCA, hereby demonstrating a new route to upcycling PET to value added products.

## 2 Materials and Methods

### 2.1 Bacterial Culture Growth and Maintenance

All bacterial strains used in this study are listed in Table 1. *P. umsongensis* GO16 was maintained on minimal salt medium (MSM) agar plates supplemented with 20 mM disodium terephthalate (TPA) as a selective carbon source (Narancic et al. 2021). All GO16 inoculums were prepared with MSM supplemented with 20 mM TPA. *E. coli* and *P. putida* KT2440 were grown on Lysogeny Broth Medium, supplemented with relevant antibiotics as necessary. MSM was prepared by adding in 1 L DI H_2_O; 9 g L^−1^ Na_2_HPO_4_.12H_2_O, 1.5 g L^−1^ KH_2_PO_4_, 1 g L^−1^ NH_4_Cl. Prior to the addition of cultures, MgSO_4_ (20 g L^−1^) and SJ Trace Elements (in 1 L DI H_2_O; 4 g L^−1^ ZnSO_4_.7H_2_0, 10 g L^−1^ FeSO_4_ .7H_2_O, 1 g L^−1^ CuCl_2_.7H_2_O, 1 g L^−1^ MnCl_2_.4H_2_O, 1 g L^−1^ Na_2_B_4_O_7_.10H_2_O, 0.2 g L^−1^ NiCl_2_.6H_2_O, 0.3 g L^−1^ Na_2_MoO_4_.2H_2_O, 20 g L^−1^ CaCl_2_, 1.2 g L^−1^ Fe(III)NH_2_. Citrate) were added in a 10^−3^ dilution, i.e., 1 mL in 1 L.

**TABLE 1:** Strains used in this study.

Strain name	Features	Reference
*E. coli* DH5α	Cloning strain and plasmid maintenance.	(Hanahan 1983)
*E. coli* DH5α λpir	Cloning strain and pKnock plasmid maintenance.	(Platt et al. 2000)
*E. coli* HB101 (PRK600)	Helper strain for tripartite conjugation.	(Kessler et al. 1992)
*P. umsongensis GO16*	Soil bacterium with capacity to metabolise TPA, EG and HMF.	(Kenny et al. 2008)
GO16 Δ*hmfF*	GO16 strain with putative FDCA decarboxylase deleted.	This study
GO16 Δ*psfG*	GO16 strain with putative HMFCA dehydrogenase deleted.	This study
GO16 Δ*psfA*	GO16 strain with putative FFCA dehydrogenase deleted.	This study
GO16 Δ*hmfT*	GO16 strain with putative HMFCA transporter deleted.	This study
GO16 Δ*hmfI*	GO16 strain with HmfI transporter protein deleted.	This study
GO16 Δ*psfG* pBT'T*‐hmfH*	GO16 Δ*psfG* overexpressing HmfH of *C. basilensis* HMF14 from the plasmid pBT'T‐*hmfH*.	This study
GO16 Δ*psfA* pBT'T*‐hmfH*	GO16 Δ*psfA* overexpressing HmfH of *C. basilensis* HMF14 from the plasmid pBT'T‐*hmfH*.	This study
GO16 GA	GO16 Δ*hmfF* overexpressing PsfG and PsfA from the plasmid pBT'T‐*psfGA*.	This study
GO16 TGA	GO16 Δ*hmfF* overexpressing HmfT, PsfG and PsfA from the plasmid pBT'T‐*hmfTpsfGA*.	This study
*P. putida* KT2440	*P. putida* mt‐2 strain cured of the TOL plasmid. Used as a biotechnological workhorse in research.	(Jiménez et al. 2002)
KT2440 G	*P. putida* KT2440 overexpressing PsfG from the plasmid pBT'T‐*psfG*.	This study
KT2440 GA	*P. putida* KT2440 overexpressing PsfG and PsfA from the plasmid pBT'T‐*psfGA*.	This study
KT2440 TGA	*P. putida* KT2440 overexpressing HmfT, PsfG and PsfA from the plasmid pBT'T‐hmfTpsfGA vector	This study

### 2.2 Generation of Deletion Mutants

Deletion of target genes in *P. umsongensis* GO16 was conducted using a modified CRISPR/Cas9 protocol (Liu et al. 2022). In brief, a pKnock suicide vector containing homology regions roughly 800‐bp upstream and downstream of the locus for deletion was generated via Gibson Assembly (New England Biolabs, UK). This plasmid was transformed into *P. umsongensis* GO16 from *E. coli* DH5α λpir via tri‐parental conjugation with the aid of the *E. coli* PRK600 helper strain. Integrants were selected for by plating on MSM‐TPA supplemented with kanamycin to a concentration of 50 μg/mL (MSM‐TPA Kan). After confirming chromosomal integration, cells were prepared for electroporation with pCas9 and plated on MSM‐TPA supplemented with kanamycin 50 μg/mL and gentamicin 30 μg/mL (MSM‐TPA KanGent) (Choi et al. 2006). Expression of Cas9 was induced by the addition of 2% L‐arabinose to the transformants in MSM‐TPA KanGent liquid culture. After 2 h, the cells were electroporated with the specific pgRNA to the gene of interest and plated overnight on MSM‐TPA supplemented with gentamicin 30 μg/mL and tetracycline 15 μg/mL (MSM‐TPA GentTet). The loss of kanamycin resistance, and therefore the deletion of the gene of interest, was confirmed by plating the colonies from MSM‐TPA GentTet to MSM‐TPA Kan. Colony PCR was carried out to preliminarily verify deletions, with full confirmation done by Sanger sequencing (Eurofins, Ireland). The deletion strains were sub‐cultured to MSM‐TA without antibiotics for up to 1 week to cure them of pCas9 and pgRNA.

Deletions of the *hmfT* and *hmfI* genes were done using a modified CRISPR/Cas3 protocol (Lammens et al. 2023). Briefly, a pSEVA237C plasmid containing 500‐bp regions upstream and downstream of the locus of interest was assembled using Gibson Assembly. A pCas3cRh plasmid containing the crRNA targeting the gene of interest was also prepared. Both plasmids were electroporated into *P. umsongensis* GO16 and plated on MSM‐TPA KanGent. Colonies were picked and used to inoculate LB broth Kan (5 mL) supplemented with L‐rhamnose, to a final concentration of 0.1% to induce the expression of the Cas3 complex enzymes. The culture was grown overnight and plated onto MSM‐TPA KanGent. Colony PCR was done on selected colonies to screen for the deletion of the target gene, followed by Sanger sequencing for confirmation (Eurofins, Ireland). The pCas3cRh and pSEVA237C plasmids were cured by the addition of the pSEVA521‐OriT plasmid, which contains crRNA targeting the remaining vectors.

Vectors, primers and gRNA used are listed in the supplementary information (Tables S1, S2 and S3, respectively).

### 2.3 Growth Study Conditions

Growth of *P. umsongensis* GO16 and derived mutants was done in MSM cultures (50 mL) supplemented with 10 mM of the desired carbon source (HMF, HMFCA, FFCA, FDCA, 2‐furoic acid, Fluorochem, UK) in 250‐mL flasks stoppered with cellulose plugs and aluminium foil. Pre‐cultures were prepared by inoculating single colonies of the strain of choice from an MSM‐TPA agar plate into MSM‐TPA (3 mL) supplemented with relevant antibiotics if necessary. These were grown overnight (12–16 h) at 200 RPM and 30°C. Each flask was then inoculated with 1 mL of overnight culture, or such that the starting OD_600_ was 0.05, and then transferred to a shaker incubator at 200 RPM and 30°C. After 48 h, the cultures were centrifuged (Eppendorf 5403R, Germany) at 6000 *xG* for 5 min. The supernatant was discarded, and the resulting pellets were resuspended in 1 mL of DI H_2_O to remove residual salts, transferred to 1.5‐mL tubes (Eppendorf, Germany) and centrifuged on a benchtop centrifuge (Eppendorf MiniSpin plus Centrifuge, Germany) at maximum speed for 5 min. The supernatant was discarded once more, and the pellets were lyophilised to obtain the cell dry weight (CDW) of the samples.

### 2.4 GO16 Δ*hmfT* Consumption Studies

To analyse the ability of GO16 Δ*hmfT* to import HMF derivatives into the cytoplasm, cells were grown in 50 mL of MSM cultures supplemented with 55 mM of glycerol in 250 mL of flasks at 30°C and 200 RPM. After overnight growth (16–18 h), the cell pellets were collected by centrifugation at 6000 *xG* for 10 min (Eppendorf 5430R, Eppendorf, Germany). The cells were concentrated to an OD_600_ of 5 or roughly 1.3 g/L of CDW and resuspended in 10 mL of MSM supplemented with 55 mM of glycerol and 10 mM of HMF in 100 mL of flasks. The flasks were incubated at 30°C and 200 RPM, with 200 μL of samples taken at specified intervals and centrifuged at max speed for 2 min (Eppendorf MiniSpin plus Centrifuge, Eppendorf, Germany). The supernatant was collected and stored for further analysis.

### 2.5 Biotransformation of HMF to FDCA Using *P. umsongensis* GO16 Mutants

Biotransformations were carried out in a 10‐mL working volume in 100‐mL flasks and modified based on existing reports (Koopman et al. 2010a). Pre‐cultures were prepared by inoculating a 1‐L flask containing 100 mL of MSM supplemented with the corresponding concentration of carbon source that equated to 2 g_C_L^−1^(i.e., 55 mM glycerol, 80 mM EG or 20 mM TPA In the case of mock PET hydrolysate, 1 g_C_L^−1^ each of TPA and EG, corresponding to 10 mM and 40 mM, respectively, was used). Overnight *P. umsongensis* GO16 Δ*hmfF* culture was added, along with 2 mM of glucose to boost initial growth. The pre‐culture was incubated for 16–20 h at 200 RPM and 30°C. In the case of EG‐grown cells, the growth period was extended to 32–40 h due to slower growth on this two carbon molecule. The cells were then pelleted at 6000 *xG* (Eppendorf) for 10 min before being washed twice with MSM modified with 5‐fold concentrated phosphate salts for improved pH buffering capacity (45 g L^−1^ Na_2_HPO_4_.12H_2_O, 7.5 g L^−1^ KH_2_PO_4_) at 6000 *xG* for 5 min. The OD_600_ was taken and the resulting cells were concentrated to OD_600_ 20 or roughly 5 g/L of CDW (OD_600_ 1 = 0.261 g/L CDW) in 10 mL of x5‐PO_4_^−3^‐MSM containing the relevant carbon source to 2 g_C_ L^−1^ and 50 mM HMF. 200 μL of samples were taken at specified intervals and centrifuged at max speed for 2 min (Eppendorf MiniSpin plus Centrifuge, Germany). The supernatant was collected and stored for further analysis.

### 2.6 *In Trans* Overexpression of *hmfH* , *hmfT, psfG* and *psfA*

To compare the activity of PsfG, PsfA, and the previously described HMF oxidising enzyme HmfH, pBT'T‐*hmfH* was constructed. The 1740 bp *hmfH* gene from *C. basilensis* HMF14 was synthesised chemically with homologous overhangs to the pBT'T vector (Twist Bioscience, USA). The obtained fragment was cloned into linearised pBT'T overexpression vector via Gibson Assembly (NEBuilder HiFi DNA Assembly Master Mix, UK) as per manufacturer's guidelines.

HmfT, PsfG and PsfA were overexpressed in GO16 Δ*hmfF* and *P. putida* KT2440 via the pBT'T overexpression vector to further characterise their roles. The three genes encoding HmfT, PsfG and PsfA lie consecutively in the *hmf* cluster and were amplified as either a 759 bp, 2216 bp or 3577 bp fragment to generate PsfG, PsfGA and HmfTPsfGA, respectively. This was done using purified *P. umsongensis* GO16 genomic DNA (GeneJET Genomic DNA Purification Kit, Fisher Scientific, UK) via PCR using primers with homologous overhangs (Merck, Ireland) to the pBT'T vector (Q5 High‐Fidelity DNA Polymerase, New England Biolabs, UK). The resulting fragments were cloned into linearised pBT'T vector using Gibson Assembly (NEBuilder HiFi DNA Assembly Master Mix, UK) as per the manufacturer's guidelines.

The resulting plasmids were transformed into chemically competent *E. coli* DH5α via heat shock and plated on LB agar plates supplemented with kanamycin 50 μg/mL (LB‐Kan). Transformed colonies were verified using colony PCR and sequencing (Eurofins, Ireland). The strains with verified constructs were grown in LB‐Kan broth (5 mL) overnight and constructs were purified using a plasmid extraction kit (GeneJET Plasmid Miniprep Kit, Fisher Scientific, UK). Purified pBT'T‐*hmfH* was electroporated into GO16 Δ*psfG* and GO16 Δ*psfA*. Purified pBT'T‐*psfG* (G), pBT'T‐*psfGA* (GA) and pBT'T‐*hmfT‐psfGA* (TGA) were electroporated into either GO16 Δ*hmfF* or *P. putida* KT2440 cultures. Transformants were screened via colony PCR and the presence of the correct plasmid was confirmed by Sanger sequencing (Eurofins, Ireland).

### 2.7 Analytical Methods

Quantification of HMF, FDCA, HMFCA and FFCA was done using an adapted HPLC method (Godoy et al. 2022). In brief, samples were diluted with DI H_2_O such that there would be no more than 1 mM of HMF/FDCA per sample vial. A 10 mM trisodium citrate buffer, equilibrated to pH 2.5 using acetic acid, was used as the mobile phase. A Zorbax 250 mM × 5 mM C18 column was equipped onto an Agilent 1260 II Infinity HPLC System, with the column temperature maintained at 30°C and pressure at approximately 150 bar. Total run time per sample was 5 min, and the observed compounds eluted at 2.9 min (FDCA), 3.4 min (HMFCA) and 3.9 min (HMF). HMF was detected at 260 nm, whereas HMFCA and FDCA were detected at 280 nm.

## 3 Results

### 3.1 Presence and Functionality of the *Hmf* Cluster in *P. umsongensis* GO16

**FIGURE 2:**
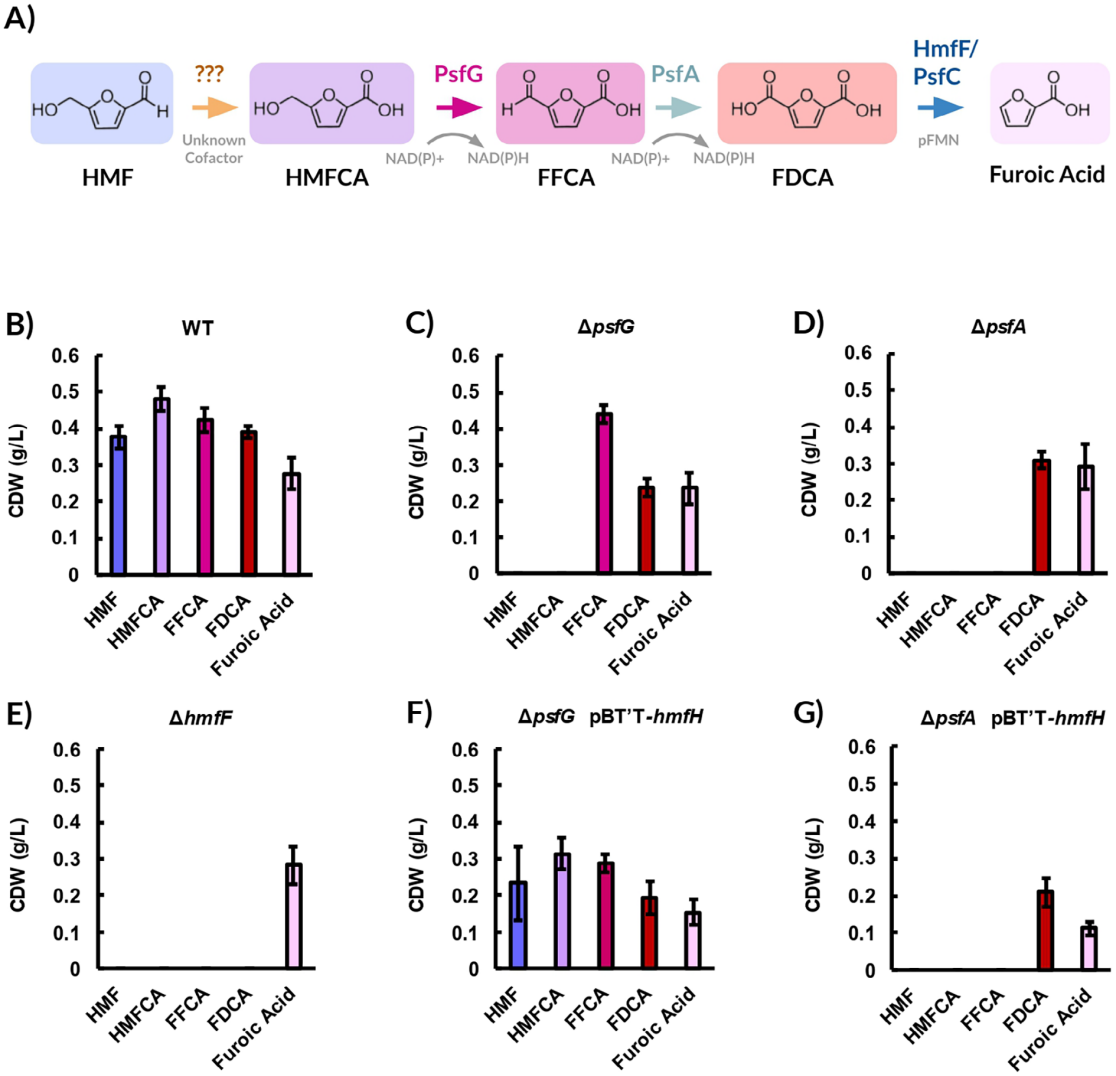
Biomass accumulated by HMF upper pathway enzymes knockout strains on 10 mM of HMF and derivatives after 48 h. (A) The HMF upper pathway in *P. umsongensis* GO16, featuring proposed enzymes for each oxidative step. PsfG and PsfA putatively utilise NAD(P) + as cofactors, while HmfF and PsfC require a prenylated flavin mononucleotide (pFMN). Biomass accumulated by (B) wild type *P. umsongensis* GO16, (C) GO16 Δ*psfG*, (D) Δ*psfA*, (E) Δ*hmfF*, (F) Δ*psfG* pBT'T‐*HmfH* and (G) Δ*psfa* pBT'T‐*hmfH* on 10 mM of HMF and derivatives after 48 h. It should be noted that furoic acid has five carbons, while the other substrates have six, and this may cause a slight variation in biomass. An OD_600_ of 1 correlates to 0.261 g CDW/L. Error bars represent standard deviation between biological replicates (*n* = 3).

In the work that characterised the *hmf* operon of *P. putida* ALS1267, it was noted that a *P. umsongensis* BS3657 strain contains a *hmf* operon with a 94.29% nucleotide identity (Crigler et al. 2020). A BLAST search revealed that *hmf* cluster (93.98% nucleotide identity, 99% query cover) was also present in *P. umsongensis* GO16's genome (Figure 1B). While it appeared that the genetic capacity to metabolise HMF was present (Table 2), GO16's ability to actually do so was not known. When GO16 was cultivated in MSM supplemented with 10 mM HMF as a sole carbon and energy source, after 48 h a biomass of 0.35 g/L on HMF was reached, illustrating that *P. umsongensis* GO16 can indeed oxidise HMF (Figure 2B). Testing other upper pathway intermediates such as FDCA (10 mM) and 2‐furoic acid (10 mM) also yields biomass, suggesting the HMF oxidation proceeds via these intermediates in GO16. It should also be noted that growth on HMF was not possible above 10 mM, highlighting the growth inhibitory effect associated with this furanic aldehyde. This toxicity has previously been alleviated by increasing the starting inoculum density, through the sheer increase in cell number and therefore detoxifying enzymes (Yu and Stahl 2008; Jönsson and Martín 2016). Indeed when a higher starting OD_600_ of 0.1 and 0.25 was used, GO16 was able to grow on 20 mM HMF, albeit to only 0.15 and 0.2 g/L of CDW, respectively (Figure S1).

**TABLE 2:** Genes of the *P. umsongensis* GO16 *hmf* cluster.

Gene	Annotated protein	Function
*hmfA*	Furoyl‐CoA oxidase	Oxidises furoyl‐CoA to 2‐oxo‐5‐furoyl‐CoA^1^
*hmfB*	Furoyl‐CoA oxidase	Aids in oxidation furoyl‐CoA to 2‐oxo‐5‐furoyl‐CoA^1^
*hmfC*	Furoyl‐CoA oxidase	Aids in oxidation furoyl‐CoA to 2‐oxo‐5‐furoyl‐CoA^1^
*hmfD*	Furoyl‐CoA synthetase	Ligates CoA moiety to 2‐furoic acid, producing furouyl‐CoA^1^
*hmfE*	2‐oxoglutaroyl‐CoA hydrolase	Cleaves 2‐oxoglutaryl‐CoA into 2‐ketoglutarate^1^
*hmfI*	Furoic acid transporter	Putatively transports lower pathway intermediates to cytoplasm^2^
*hmfF*	FDCA decarboxylase	Decarboxylates FDCA to 2‐furoic acid^1^
*hmfT*	HMFCA transporter	Transports HMFCA from periplasm to cytoplasm^2^
*psfG*	HMFCA dehydrogenase	Oxidises HMFCA to FFCA^3^
*psfA*	FFCA dehydrogenase	Oxidises FFCA to FDCA^3^
*psfB*	LysR‐family transcriptional regulator	Regulator of upper pathway genes^1^
*psfC*	HmfF accessory protein	Produces flavin mononucleotide cofactor of HmfF^2^
*hyd*	Putative 5‐hydroxy‐2‐furoyl‐CoA hydrolase	Putatively cleaves furan ring of 5‐hydroxy‐2‐furoyl‐CoA^2^
*psfD*	Furoyl‐CoA oxidase accessory protein	Inserts molybdopterin cofactor into HmfA^2^
*araC*	Helix‐turn‐helix transcriptional regulator	Regulator of lower pathway genes^2^

*Note:* Functions ascertained based on annotations by (Koopman et al. 2010b)^1^, (Donoso et al. 2021)^2^ or this work^3^.

### 3.2 PsfG, PsfA and HmfF Are Involved in the HMF Upper Pathway

To allow for the accumulation of FDCA, the upper pathway in GO16 has to be defined, and the through‐conversion into the TCA cycle intermediate 2‐ketoglutarate intercepted. Despite showing high similarity to the well characterised *hmf* operon of *C. basilensis* HMF14, no homologues to the enzymes required for HMF oxidation to FDCA are present in the GO16 *hmf* cluster. The identity of these enzymes is critical for further strain engineering with FDCA production in mind. In recent work carried out in *P. putida* KT2440 and *P. taiwanensis* VLB120, the role of the periplasmic aldehyde oxidoreductase complex PaoEFG was highlighted in the context of HMF oxidation to HMFCA (Lechtenberg et al. 2024b). *P. umsongensis* GO16 lacks homologues to the three genes that encode for PaoEFG but does possess a number of other periplasmic aldehyde dehydrogenase encoding clusters, which presumably fulfil the same role. *Pseudomonas* sp. are remarkably adept at tolerating oxidative stress and their repertoire of non‐specific oxidases and dehydrogenases has been theorised to play a role in this robustness (Kim and Park 2014). It should be noted that neither *P. putida KT2440* nor *P. taiwanensis* VLB120 have the capacity to natively oxidise HMFCA into FFCA and FDCA, so we sought to find which enzymes in *P. umsongensis* GO16 conferred this ability to it.

Looking at the *P. umsongensis* GO16 *hmf* cluster, many genes are homologous to those found in the *hmf* operon of *C. basilensis* HMF14. One major difference, however, is the absence of *hmfH*, encoding for HMF oxidoreductase. Previously thought to carry out the three consecutive oxidations of HMF to FDCA, it now appears that its primary substrate is HMFCA, converting it into FFCA. Heterologous expression of this gene in *P. putida* S12 allowed for the production of FDCA in this strain (Koopman et al. 2010a). However, the lack of specialised enzymes which carry out such an important step in the GO16 HMF pathway seems improbable. Two candidates potentially fulfil this role in GO16. PsfG (F6476_07500), a putative short chain alcohol dehydrogenase, and PsfA (F6476_07505), a putative NAD+ dependent aldehyde dehydrogenase, are situated within the *hmf* cluster (Table 2, Figure 1B). Both enzymes were first identified in *P. putida* Fu1, which can metabolise furfuryl alcohol, an analogue of HMF (Nichols and Mertens 2008). PsfA is implicated in the oxidation of furfuryl alcohol to furfural, while PsfG putatively oxidises furfural to 2‐furoic acid. These enzymes were speculated to act in the oxidation of HMF as well as furfuryl alcohol, but this was not experimentally validated (Donoso et al. 2021). Indeed, it had been noted that the genomes of HMF metabolising bacteria contained either a copy of HmfH or PsfG, but never both, suggesting analogous activity between the two enzymes (Donoso et al. 2021).

To ascertain the putative roles of PsfA and PsfG in HMF upper pathway oxidation, the genes encoding these two enzymes were deleted. GO16 Δ*psfA* and GO16 Δ*psfG* both fail to grow on 10 mM HMF and HMFCA as a sole carbon source (Figure 2C,D). When 10 mM of FFCA was used, GO16 Δ*psfA* failed to grow once more, but GO16 Δ*psfG* reached 0.44 g/L of biomass. When 10 mM of FDCA is used, however, GO16 Δ*psfA* and GO16 Δ*psfG* achieve 0.31 g/L and 0.24 g/L of CDW, respectively. Using 10 mM of 2‐furoic acid also results in growth, at 0.24 g/L for GO16 Δ*psfA* and 0.29 g/L for GO16 Δ*psfG*. Based on these results, it would appear that PsfG and PsfA's substrates lie in the oxidation steps preceding FDCA (Figure 2A). The conversion of HMFCA to FFCA is carried out by PsfG, as GO16 Δ*psfG* fails to grow on any substrate up to HMFCA (Figure 2C). Similarly, the conversion of FFCA to FDCA is carried out by PsfA, as GO16 Δ*psfA* fails to grow on any substrate up to FFCA. The recovered growth of GO16 Δ*psfG*‐pBT'T‐*hmfH* on HMF (0.23 g/L) and HMFCA (0.31 g/L), but not for GO16 Δ*psfA* pBT'T‐*hmfH*, is consistent with the postulated analogous roles of HmfH and PsfG in oxidising HMFCA to FFCA (Figure 2F,G).

Previous work has identified HmfF and PsfCas as the two enzymes responsible for the decarboxylation of FDCA to 2‐furoic acid (Koopman et al. 2010b). These belong to the UbiD/UbiX type system, where UbiD acts as the decarboxylase and UbiX is a prenyltransferase which generates a prenylated flavin mononucleotide cofactor necessary for efficient decarboxylation of target molecules (Marshall et al. 2017). When *hmfG*, showing 96% identity to *psfC* of *P. umsongensis* GO16, from *C. basilensis* HMF14 was heterologously expressed in *P. putida* S12, no 2‐furoic acid formed when the cells were incubated with FDCA. However, slight decarboxylation activity was noted when *hmfF* was heterologously expressed (Koopman et al. 2010b). With this in mind, *hmfF* (F6476_07490) was the next gene deletion target. GO16 *ΔhmfF* fails to grow on HMF or FDCA but achieves 0.28 g/L of biomass when 10 mM of 2‐furoic acid is supplied as the sole carbon source (Figure 2E). As this strain is unable to convert FDCA to 2‐furoic acid, the conversion of FDCA to further metabolism was disrupted and its accumulation was now theoretically possible.

### 3.3 HmfT Is Responsible for Cytoplasmic Uptake of HMFCA

**FIGURE 3:**
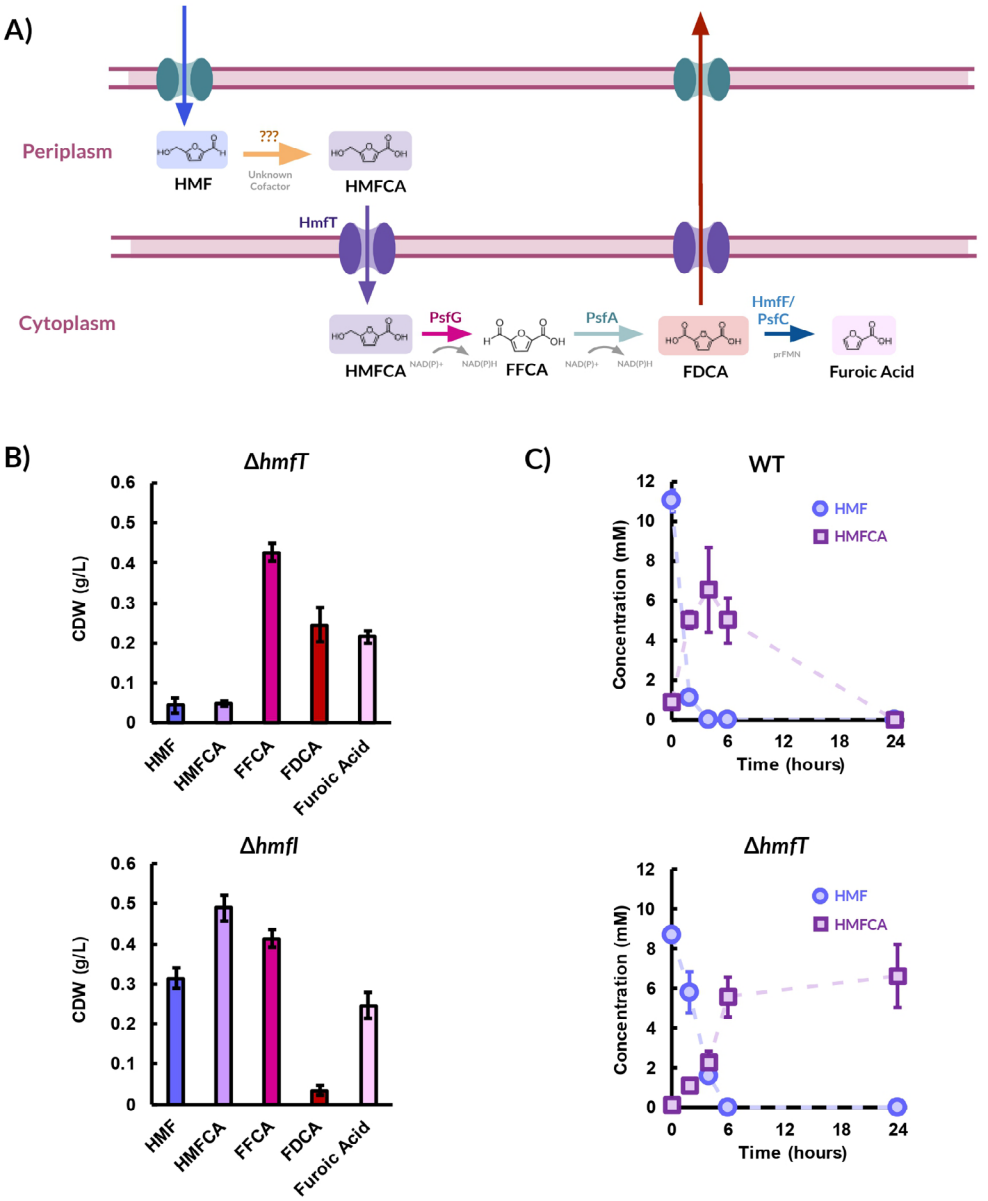
Uptake of HMFCA from the periplasm to the cytoplasm by HmfT. (A) The proposed route for HMF oxidation in *P. umsongensis* GO16. (B) Growth of strains GO16 *ΔhmfT* and GO16 *ΔhmfI* on 10 mM HMF and its derivatives after 48 h. (C) The consumption profiles of HMF and HMFCA by GO16 WT and GO16 *ΔhmfT* grown on 55 mM glycerol within 24 h. Error bars represent standard deviation between biological replicates (*n* = 3).

The predicted pathway for FDCA production in GO16 likely includes the initial oxidation to HMFCA in the periplasm. This is followed by transporter‐mediated uptake into the cytoplasm, where PsfG and PsfA consecutively oxidise it to FFCA and finally FDCA (Figure 2A). As noted above, other *Pseudomonas* sp. display the ability to oxidise HMF to the less toxic HMFCA via periplasmic aldehyde oxidoreductases. However, the proteins in the *hmf* cluster of *P. umsongensis* GO16 are all predicted by pSORTdb (Lau et al. 2021) to localise in either the cytoplasm or cytoplasmic membrane. It would therefore stand to reason that HMFCA has to be imported into the cytoplasm by a specific transporter to be further metabolised. Within the *hmf* cluster lie two transporters, HmfI (F6476_RS07485) and HmfT (F6476_RS07495). HmfT shares 58% sequence identity to the HmfT1 transporter of *C. basilensis* HMF14, recently described as an HMFCA transporter when expressed in *P. putida* KT2440 and *P. taiwanensis* VLB120 (Lechtenberg et al. 2024b). In contrast, no previously characterised homologues to HmfI have been described. To ascertain the putative roles of these transporters in the HMF upper pathway, the genes were deleted, and the resulting strains were screened for growth on HMF and its derivatives (Figure 3). GO16 Δ*hmfT* was able to accumulate biomass when grown on FFCA, FDCA and furoic acid comparable to the wild type. However, GO16 Δ*hmfT* achieved approximately 10‐fold lower biomass compared to the wild type when grown on HMF (0.04 g of CDW/L vs. 0.38 g of CDW/L) and HMFCA (0.05 g of CDW/L vs. 0.048 g of CDW/L), respectively (Figures 2B and 3B). These results implicate HmfT in the transport of HMFCA from the periplasm in *P. umsongensis* GO16. GO16 Δ*hmfI* was able to grow on all HMF derivatives to comparable levels as the wild type, except for FDCA, accumulating only 0.03 g of CDW/L on it (Figure 3B). Specific transporters for FDCA are scantly described in literature but *Pseudomonas* sp. ALS1279, which possesses many of the genes from the *P. umsongensis* GO16 operon with the notable exception of *hmfI*, has previously been described (Donoso et al. 2021). This strain was able to grow on all HMF derivatives up to furoic acid, except for FDCA, with the authors speculating it may be due to the absence of specific transporters for the dicarboxylic acid. HmfI may therefore be one such transporter, based on the failure of GO16 Δ*hmfI* to grow on FDCA. It should be noted that while growth was severely hindered, both transporter knockouts were still able to accumulate detectable levels of biomass when grown on their putative substrates. This seems to imply that non‐specific transporters are capable of taking up these substrates, albeit at significantly reduced efficiency.

To further highlight HmfT's transporter capabilities, HMF consumption studies were carried out. *P. umsongensis* GO16 WT and GO16 Δ*hmfT* were grown on 55 mM of glycerol to approximately 1 g/L of CDW and challenged with 10 mM HMF. In the WT cultures, HMF is quickly depleted, followed by the transient accumulation of HMFCA, which also becomes depleted within 24 h (Figure 3C). In GO16 Δ*hmfT* HMFCA level persists over 24 h, showing the role of HmfT in the uptake of HMFCA from the periplasm to the cytoplasm in *P. umsongensis* GO16.

### 3.4 GO16 *ΔhmfF* Biotransforms HMF to FDCA

**FIGURE 4:**
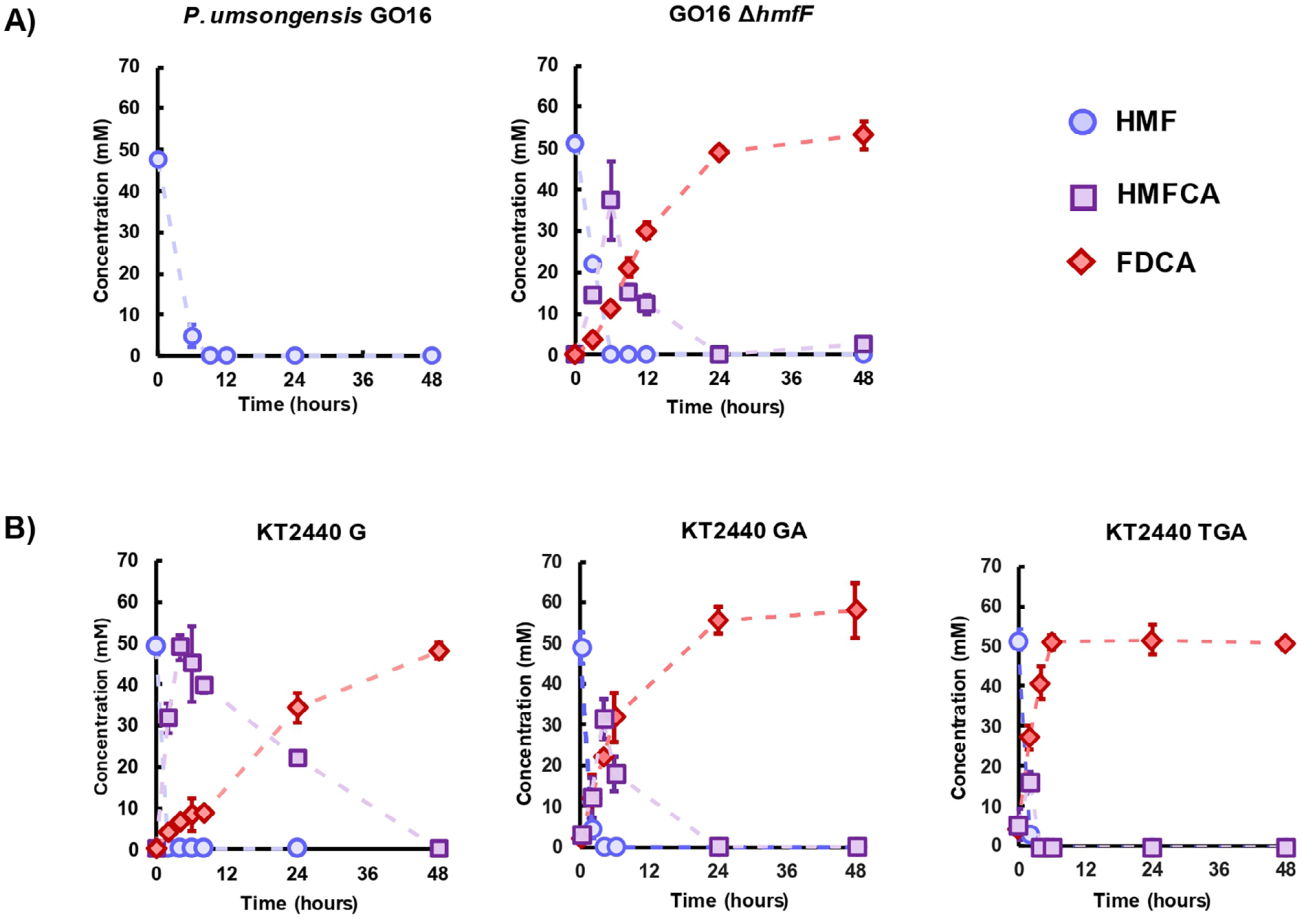
Conversion of 50 mM HMF to FDCA using 55 mM glycerol to support growing cells biotransformation. (A) Biotransformation by *P. umsongensis* GO16 WT and GO16 Δ*hmfF* of 50 mM HMF. (B) Biotransformation by KT2440 G, KT2440 GA and KT2440 TGA of 50 mM HMF. Error bars represent standard deviation of the mean between biological replicates (*n* = 2).

GO16 *ΔhmfF* was employed in a whole cell biotransformation to test if the deletion of *hmfF* alone would be sufficient to facilitate FDCA accumulation. Growing cells were used for the biotransformations to allow for in situ enzyme regeneration and cofactor recycling, necessary for an efficient process (Wierckx et al. 2015). When GO16 *ΔhmfF* cells grown with glycerol were concentrated and used as a biocatalyst in the biotransformation of 50 mM HMF, complete depletion of the furanic aldehyde occurred after 6 h. This was accompanied by the accumulation of HMFCA, with a sharp increase to 37 mM after 6 h, followed by its conversion into FDCA over the next 18 h. After 24 h, only FDCA was detected, suggesting completion of the oxidation reaction (Figure 4A). This demonstrates that GO16 *ΔhmfF* can be used as a biocatalyst for the production of FDCA. In contrast, when grown on glycerol and challenged with 50 mM HMF, wild type *P. umsongensis* GO16 fully consumes the furanic aldehyde, with no build‐up of intermediates, suggesting complete metabolism (Figure 4A). It should be noted that at certain sampling time points, the mass balances suggest there is ‘missing’ carbon that our detection method cannot account for. As well as oxidative detoxification, bacteria can also reduce HMF to the alcohol 2,5‐bis(hydroxymethyl)furan (Lechtenberg et al. 2024a; Hossain Gazi et al. 2017). We were unable to detect this molecule, so we cannot rule out the possibility that GO16 also detoxifies HMF to its alcohol form.

### 3.5 Heterologous Expression of *psfG, psfA* and *hmfT* Allows *P. putida* KT2440 to Efficiently Produce FDCA

To further validate the roles of PsfA and PsfG in the oxidations of HMFCA to FDCA, their genes were heterologously expressed in *P. putida* KT2440. As noted previously, *P. putida* KT2440 has the capacity to natively oxidise HMF to HMFCA and FFCA to FDCA, primarily by PaoEFG and supported by cytoplasmic aldehyde oxidases such as AldB‐I (Lechtenberg et al. 2024b). This strain, however, cannot natively convert HMFCA to FFCA. When PsfG was overexpressed in *P. putida* KT2440 from the pBT'T vector, and using the same strategy as with GO16 Δ*hmfF*, KT2440 G cells grown on glycerol were able to convert 50 mM of HMF to 50 mM FDCA in 48 h, with a specific productivity of 320 ± 42 μmol FDCA/gCDW/h (Figure 4B). Metabolism of HMFCA was slow, however, with 22 mM still present after 24 h. In previous work using *P. putida* S12, heterologous expression of *hmfT1*, the HMFCA transporter found in the *C. basilensis* HMF14 *hmf* operon, and *adh*, showing 63% sequence similarity to the *psfA* gene of *P. putida* Fu1, along with *hmffH*, drastically improved FDCA production with low intermediate accumulation (Wierckx et al. 2015; Pham et al. 2020). As the *P. umsongensis* GO16 *hmf* cluster contains *hmfT* and *psfA*, both were natural targets for overexpression and HMFCA de‐bottlenecking. Thus, pBT'T‐*psfGA*, overexpressing both dehydrogenases, and pBT'T‐*hmfTpsfGA*, overexpressing both dehydrogenases and the HMFCA transporter, were constructed. These were used to generate KT2440 GA and KT2440 TGA, which showed significantly improved productivity compared to KT2440 G at 662 ± 124 μmol and 794 ± 31 μmol of FDCA/gCDW/h, respectively (Figure 4B). Noticeably, KT2440 TGA only accumulated 16 mM of HMFCA at 6 h, which was rapidly consumed after. This reduction in intermediate accumulation seems to indicate that the uptake of HMFCA is the rate‐limiting step in FDCA production, rather than insufficient enzyme activity.

### 3.6 Upcycling of PET Monomers Into FDCA

**FIGURE 5:**
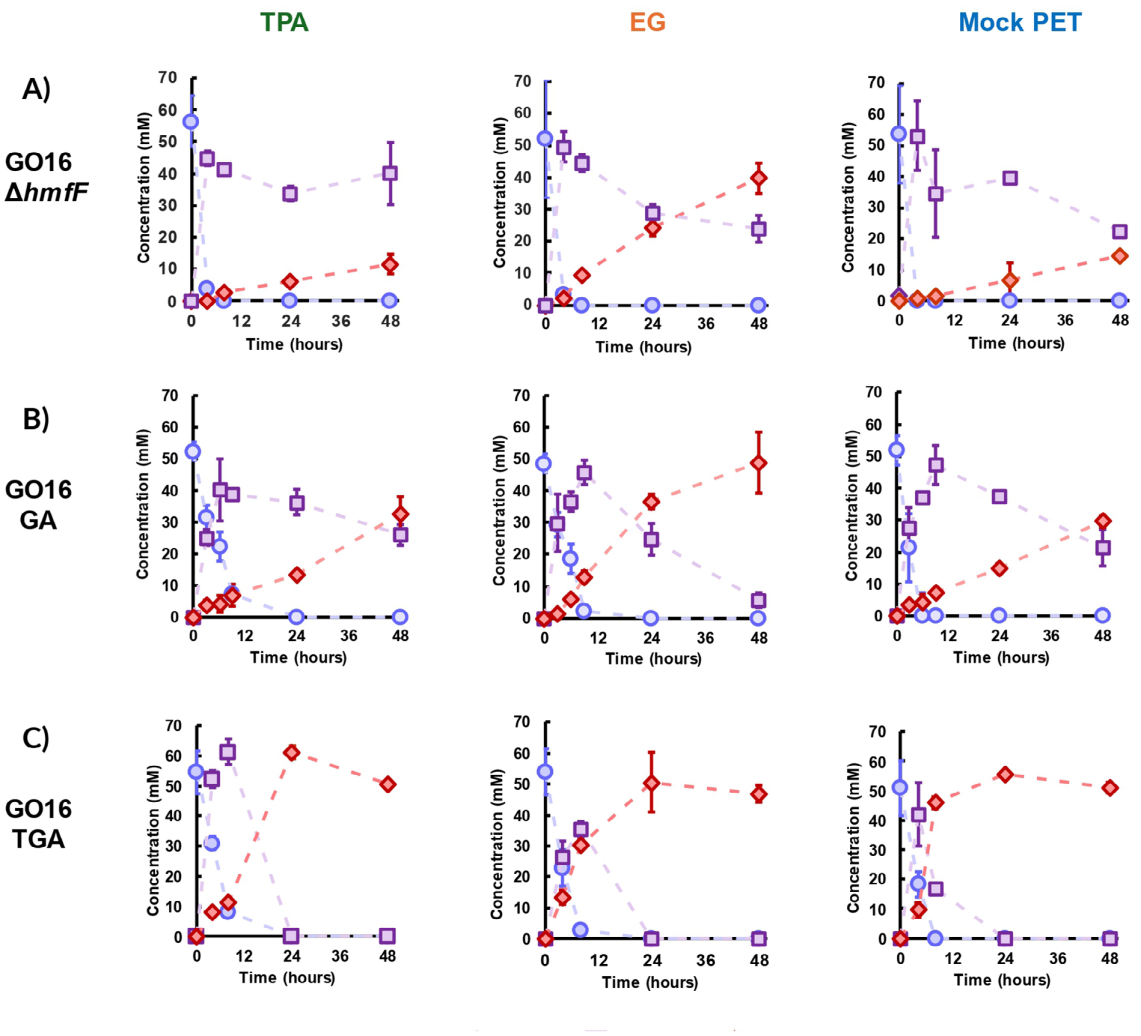
Production of FDCA from *P. umsongensis* GO16 derived strains grown on PET monomers. (A) GO16 Δ*hmfF* shows hindered capacity to oxidise HMF into FDCA on terephthalic acid (TPA), ethylene glycol (EG) and mock PET hydrolysate (Mock PET). (B) Overexpression of *psfGA* improves HMF conversion to FDCA. On TPA and mock PET hydrolysate conditions, HMFCA still persists after 48 h. (C) The *in trans* overexpression of the *hmfTpsfGA* synthetic cluster significantly improves FDCA conversion on all three growing conditions. Error bars represent standard deviation of the mean between biological replicates (*n* = 2).

*P. umsongensis* GO16 is notable in its ability to consume both TPA and EG as carbon and energy sources to achieve high biomass, with these traits previously exploited to demonstrate the further valorisation potential of GO16 (Narancic et al. 2021). When grown on 80 mM EG, GO16 Δ*hmfF* is able to convert 50 mM HMF into 39 mM FDCA after 48 h (Figure 5A). While not as efficient as glycerol‐grown cells, this is presumably due to reduced metabolic efficiency and reduced equivalents used in the oxidation of the two carbon substrates (Franden et al. 2018; Li et al. 2019). When TPA‐grown cells were used, HMF was still rapidly depleted, down to 4 mM in 4 h. The conversion efficiency to FDCA was markedly reduced, however, to only 22% after 48 h, corresponding to 11 mM of the desired product (Figure 5A). HMFCA persisted throughout the timeframe, showing minimal oxidation. On EG and TPA, the consumption rate of HMFCA was 11‐fold (100 μmol gCDW h^−1^) and 17‐fold (63 μmol gCDW h^−1^) lower, respectively, compared to glycerol‐grown biotransformations (1103 μmol gCDW h^−1^). This inhibitory effect on FDCA production was also observed when a ‘mock’ PET hydrolysate consisting of equimolar amounts of TPA and EG was used (Figure 5A).

Noting the accumulation of HMFCA, we identified this as the rate‐limiting step in HMF oxidation to FDCA. Initially, we sought to alleviate this bottleneck by overexpressing PsfG and PsfA, seeing as they are the enzymes involved in the oxidations downstream of HMFCA in *P. umsongensis* GO16. Thus, GO16 Δ*hmfF* was transformed with the pBT'T‐PsfGA vector, generating GO16 GA (Table 1). GO16 GA showed improved FDCA productivity when grown on TPA (80 μmol gCDW h^−1^), EG (169 μmol gCDW h^−1^) and mock PET hydrolysate (60 μmol gCDW h^−1^), indicating a beneficial effect of the overexpression of the two dehydrogenases (Figure 5B). EG‐grown GO16 GA showed near total HMF to FDCA conversion after 48 h, with only 5 mM of HMFCA left. However, GO16 GA grown on TPA and mock PET still failed to fully convert HMF to FDCA after 48 h, accumulating 26 mM and 21 mM of HMFCA, respectively (Figure 5B).

Based on the results with *P. putida* KT2440, where the best productivity was observed with overexpression of HmfT along with the two dehydrogenases, we transformed the previously generated pBT'T‐HmfTPsfGA plasmid into GO16 Δ*hmfF* to generate GO16 TGA. This conferred the biocatalyst with the capacity to fully convert 50 mM of HMF into FDCA in 24 h in a TPA powered biotransformation (Figure 5C). This represents a 12‐fold improvement in specific productivity when compared to GO16 Δ*hmfF* (Table 3). Similarly, GO16 TGA cells grown on EG and mock PET hydrolysate showed 5‐fold and 19‐fold increases in specific productivity, respectively. The results for mock PET hydrolysate powered strains are particularly interesting, as when in the presence of another carbon source (in this case TPA), EG can likely act as an energy substrate. This yields even more reducing equivalents on top of the ones generated from HMF oxidation, generating more cellular power and potentially a more efficient biocatalyst (Li et al. 2019). This presents now the potential to use GO16 as a chassis for the upcycling of PET hydrolysate as a growth source to power the production of FDCA. Furthermore, GO16 GA and TGA cells grown on glycerol were tested for their FDCA producing capabilities, reaching specific productivities of 411 ± 10 and 767 ± 24 μmol of FDCA g CDW^−1^, respectively (Table 3 and Figure S2) Thus, GO16 TGA provides a platform to upcycle different waste derived feedstocks, such as waste glycerol and PET hydrolysate into FDCA.

**TABLE 3:** FDCA production rate (μmol FDCA per hour per g of CDW.) of GO16 Δ*hmfF*, GO16 TGA and GO16 GA on different carbon sources.

Strain	Growth substrate	Biomass concentration (g CDW L^−1^)	Productivity (μmol FDCA g CDW^−1^ h^−1^)	Source
*Pseudomonas putida* S12	40 mM Glycerol	15	276 ± 89	(Koopman et al. 2010a)
*Pseudomonas putida* S12	60 mM Glycerol, 1 g/L yeast extract	11.2	727	(Pham et al. 2020)
*Raoultella ornitholytica* BF60	Terrific Broth	OD_600_: 100	41	(Yuan et al. 2018)
*Mycobacterium* sp. MS 160	5 g/L glycerol, 2 g/L sodium acetate, 10 g/L yeast extract	N/A	500 μM/h^a^	(Sayed et al. 2019)
*Escherichia coli* HMFOMUT	Terrific Broth	5	21^b^	(Wang et al. 2020)
GO16 Δ*hmfF*	55 mM Glycerol 20 mM TPA 80 mM EG Mock PET hydrolysate^c^	5 (for all substrates)	388 ± 29 32 ± 4 127 ± 15 40 ± 15	This work
GO16 GA	55 mM Glycerol 20 mM TPA 80 mM EG Mock PET hydrolysate	5 (for all substrates)	411 ± 10 80 ± 22 169 ± 5 60 ± 12	This work
GO16 TGA	55 mM Glycerol 20 mM TPA 80 mM EG Mock PET hydrolysate	5 (for all substrates)	767 ± 24 415 ± 17 509 ± 21 768 ± 34	This work

^a^ Estimated due to lack of biomass data.

^b^ Estimated due to lack of time course graphs.

^c^ 10 mM TPA and 40 mM EG.

## 4 Discussion

With production of plastics breaching 400 million tonnes in 2022 and increasing year on year, the appropriate management of these polymers is vital to mitigate our impact on the planet's environments and ecosystems (OECD 2022). While mechanical recycling of PET is being increasingly adopted globally, iterative rounds of recycling shorten chain length, yielding recyclate with properties unfavourable for further packaging applications (Schyns and Shaver 2021). Nevertheless, this unusable PET can be depolymerised into its constituent TPA and EG monomers, either enzymatically (Tournier et al. 2020; Sonnendecker et al. 2022; Lu et al. 2022), chemically (Barnard et al. 2021), or via pyrolysis (Yoshioka et al. 2004; Brems et al. 2011). *Pseudomonas* species represent a promising means to upcycle theses waste monomers, due to their versatile metabolism, amenability to genetic manipulation and tolerance to abiotic stressors (Nikel et al. 2016; Bitzenhofer et al. 2021; Martin‐Pascual et al. 2021). Indeed, *P. umsongensis* GO16 has already been harnessed as a platform to convert TPA obtained through PET pyrolysis to PHAs (Kenny et al. 2008, 2012), or hydrolysed PET to PHA and HAAs (Tiso et al. 2021; Cerrone et al. 2023). Parallel biotechnology‐based strategies utilising bacterial strains to upcycle PET to vanillin, β‐ketoadipic acid and lycopene have also been demonstrated (Sadler and Wallace 2021; Werner et al. 2021; Diao et al. 2023). By identifying and intercepting the native HMF catabolic pathway in GO16, we can now add FDCA to the repertoire of valuable molecules produced by Pseudomonads using PET hydrolysate.

A challenge in PET‐monomer utilisation to obtain the biocatalyst biomass and power the HMF to FDCA bioconversion is an inhibitory effect exhibited in the presence of TPA. While EG‐grown cells (127 μmol gCDW^−1^ h^−1^) showed three‐fold lower productivity compared to those done with glycerol (388 μmol gCDW^−1^ h^−1^), the productivity of TPA‐powered biotransformation was 12‐fold lower (32 μmol gCDW^−1^ h^−1^). While productivity was improved with *the trans* overexpression of PsfG and PsfA, HMFCA persistence was still observed in TPA‐containing biotransformations. Only the overexpression of HmfT alleviated this bottleneck, suggesting hindered HMFCA import is particularly pronounced when TPA is used as a growth substrate.

The recently identified sequence of events in HMF oxidation, where HMF is oxidised to HMFCA in the periplasm, followed by transporter‐mediated uptake to the cytoplasm (Lechtenberg et al. 2024b), may suggest that TPA and HMFCA, both small, hydrophobic aromatic molecules, are competing for HmfT‐mediated transport. However, when the biotransformation was repeated with TPA‐grown cells with HMF only, the result was comparable to the biotransformation where TPA was added as a supplement for the biotransformation (Figure S3). This seems to indicate that this issue was perhaps due to the metabolic state of the cell rather than the transport challenge. Further characterisation of this inhibition phenomenon in GO16 would be of interest for the development of a more efficient biocatalyst.

Our results agree with recent literature regarding the subcellular partitioning of HMF oxidation in *Pseudomonas* species, namely the uptake of HMFCA, not HMF, from the periplasm to the cytoplasm (Lechtenberg et al. 2024b). Aldehyde toxicity is a well‐described phenomenon, due to the potential of these electrophilic species to form protein adducts and bind to DNA and proteins indiscriminately to disrupt native cell processes. Indeed, *C. basilensis* HMF14 was first investigated not for its ability to produce FDCA but rather as a form of bio‐abatement to detoxify chemically treated wood residues of HMF (Koopman et al. 2010b). With this toxicity effect in mind, it would make sense for GO16 and other *Pseudomonas* species to carry out initial oxidation of HMF to HMFCA in the periplasm. While it appears clear that PsfA and PsfG are essential for HMFCA oxidation to FDCA in GO16, the identity of the enzyme involved in HMF to HMFCA oxidation is still to be deciphered. In *P. taiwanensis* VLB120 and KT2440, the PaoEFG periplasmic aldehyde oxidoreductase complex consisting of 2Fe‐2S protein, molybdopterin‐binding subunit, and a cytochrome c, is responsible for detoxifying HMF to HMFCA (Lechtenberg et al. 2024b). While no such proteins of a suitable similarity were found in GO16, this strain does possess a myriad of other oxidoreductase clusters which may fulfil this role that warrant further research.

Our work with the deletions of the two transporters in the *hmf* operon appears to have identified HmfI as a putative FDCA transporter. While the focus of this work is on FDCA production, understanding how FDCA is taken up and metabolised is also of interest in the potential management of this bioplastic monomer. Despite its role in the uptake of FDCA, FDCA is present in the supernatant after 48 h even when the reaction fully finishes within 24 h, suggesting it is preferentially kept out of the cell.

Enzymes involved in the specific production of FDCA are scantly described in literature. The ones that are described appear to be oxidoreductases, requiring FAD cofactors that generate H_2_O_2_ upon reoxidation (Koopman et al. 2010b; Dijkman and Fraaije 2014). To our knowledge, PsfA and PsfG are the first dehydrogenases identified to be involved in the specific oxidation of HMFCA to FDCA. This presents an advantage as they generate reduced electron carriers, rather than harmful H_2_O_2_. Our FDCA specific productivity values compare favourably with the best values for FDCA whole cell biotransformations reported in literature (Table 3). Nevertheless, it would be interesting to make direct comparisons in FDCA biotransformation efficiency between an HMFCA oxidoreductase (e.g., HmfH) versus PsfG expressing strain to determine if the reduced equivalents generated are better for the cell.

Biologically produced FDCA is relatively easy to purify, as it will crash out of solution by acidifying the media, with increased purity possible via solvent extraction (Koopman et al. 2010a). Most FDCA production routes rely on heterologous expression of genes in microbial chassis such as *P. putida* S12, *G. oxydans* , or *E. coli* (Pham et al. 2020; Sayed et al. 2019; Wang et al. 2020). Other routes to FDCA production rely on in vitro cascade reactions, using a medley of oxidases from different sources (Mckenna et al. 2015, 2017; Wu et al. 2020; Tan et al. 2020; Yang et al. 2023). Utilising a native HMF metabolic pathway for FDCA production has been done previously with *R. ornothilytica* BF60 (Hossain Gazi et al. 2017). Similar to this work, the FDCA decarboxylase gene, *dcaD*, was disrupted to prevent through‐conversion of FDCA to further metabolism. Our whole cell biotransformation is unique amongst these strategies in that it allows for the upcycling of a waste feedstock PET monomers. By growing GO16 on TPA and EG and using the resulting biomass to convert HMF to FDCA, this offers a two‐pronged approach to limiting the spread of fossil plastics and aiding in the realisation of a circular economy (Narancic and O'connor 2017).

## Author Contributions

**Rhys Orimaco:** conceptualization, methodology, investigation, writing – original draft, validation. **Pauric Donnelly:** investigation. **Seán Sexton:** investigation. **Aoife McLoughlin:** investigation. **Sophie Kelly:** investigation. **Kevin E. O'Connor:** writing – review and editing, funding acquisition, validation. **Nick Wierckx:** writing – review and editing, methodology. **Tanja Narančić:** conceptualization, methodology, validation, writing – review and editing, funding acquisition, supervision.

## Conflicts of Interest

The authors declare no conflicts of interest.

## Supporting information


Data S1.

## Data Availability

The data that support the findings of this study are available from the corresponding author upon reasonable request.
