# Supplementary material for: Characterisation and Harnessing of 5‐Hydroxymethylfurfural Metabolism in Pseudomonas umsongensis GO16 for the Production of 2,5‐Furandicarboxylic Acid
Source: Microb Biotechnol. 2025 May 10;18(5):e70159. doi: 10.1111/1751-7915.70159 (PMC12064950; doi:10.1111/1751-7915.70159)
Supplement: Supplementary file 1 — Data S1. [file MBT2-18-e70159-s001.docx]

**Characterisation and Harnessing of 5-Hydroxymethylfurfural Metabolism in *Pseudomonas umsongensis* GO16 for the Production of 2,5-Furandicarboxylic Acid**

Rhys Orimaco^1,2^, Pauric Donnelly^1,2^, Seán Sexton^1^, Aoife McLoughlin^1^, Sophie Kelly^1^ Kevin E. O’Connor^1,2^, Nick Wierckx^3^, Tanja Narancic^1,2^

^1^ UCD Earth Institute and School of Biomolecular and Biomedical Science, University College Dublin, Belfield, Dublin 4, Ireland

^2^ BiOrbic - Bioeconomy Research Centre, Ireland, University College Dublin, Belfield, Dublin 4, Ireland

^3^ Institute of Bio- and Geosciences IBG-1: Biotechnology, Forschungszentrum Jülich, 52425 Jülich, Germany

***Corresponding author:** Tanja Narančić

UCD O'Brien Centre for Science

University College Dublin

Belfield D4, Ireland

**Telephone:** +353 (01) 716 2679

**Email:** [tanja.narancic@ucd.ie](mailto:tanja.narancic@ucd.ie)

**Table S1:** List of plasmids used

| **Name** | **Purpose and relevant components** | **Reference** |
| --- | --- | --- |
| pKnock | Repair template for CRISPR/Cas9 genome editing; Kan^R^, ori R6K | (Liu et al., 2022) |
| pCas9 | Contains Cas9 endonuclease and λred recombinases; Gent^R^, Cas9 endonuclease, AraC regulator | (Liu et al., 2022) |
| pgRNA | Contains 20 bp gRNA that targets gene of interest; Tet^R^ | (Liu et al., 2022) |
| pBT’T | Overexpression vector for genes of interest;  Kan^R^, pBBR1 ori, pBBR rep, P_tac,_ RBS | (Koopman et al., 2010) |
| pSEVA237C | Repair template for CRISPR/Cas3 genome editing; Kan^R^, pBBR1 rep, OriT | (Silva-Rocha et al., 2013) |
| pCas3cRh | Contains Cas3 complex nucleases and 34 bp crRNA that targets gene of interest; Gent^R^, oriT | (Csörgő et al., 2020, Lammens et al., 2023) |
| pSEVA521-OriT | Contains 34 bp crRNA that targets the OriT of the three plasmids in the CRISPR/Cas3 system, thereby self-curing themselves from the bacteria; Tet^R^, oriT crRNA | (Lammens et al., 2023) |
| pBT’T-*psfG* | pBT’T which overexpresses HMFCA dehydrogenase (*psfG*) of *P. umsongensis* GO16, | This study |
| pBT’T-*psfGA* | pBT’T which overexpresses HMFCA dehydrogenase (*psfG*) and FFCA dehydrogenase (*psfA*) of *P. umsongensis* GO16, | This study |
| pBT’T-*hmfTpsfGA* | pBT’T which overexpresses HMFCA transporter (*hmfT*) HMFCA dehydrogenase (*psfG*) and FFCA dehydrogenase (*psfA*) of *P. umsongensis* GO16, | This study |
| pBT’T-*hmfH* | pBT’T which overexpresses HMFCA oxidoreductase (*hmfH*) of *C. basilensis* HMF14, | This study |
| pKnock-HmfF | pKnock with homology arms upstream and downstream of *hmfF* for its deletion. | This study |
| pgRNA-HmfF | pgRNA with the 20 bp gRNA for targeting *hmfF*. | This study |
| pKnock-PsfG | pKnock with homology arms upstream and downstream of *psfG* for its deletion. | This study |
| pgRNA-PsfG | pgRNA with the 20 bp gRNA for targeting *psfG*. | This study |
| pKnock-PsfA | pKnock with homology arms upstream and downstream of *psfA* for its deletion. | This study |
| pgRNA-PsfA | pgRNA with the 20 bp gRNA for targeting *psfA*. | This study |
| pSEVA237C-HmfT | pSEVA237C with the homology arms upstream and downstream of *hmfT* for its deletion. | This study |
| pCas3cRh-HmfT | pCas3cRh with the 34 bp crRNA for targeting *hmfT*. | This study |
| pSEVA237C-HmfI | pSEVA237C with the homology arms upstream and downstream of *hmfI* for its deletion. | This study |
| pCas3cRh-HmfI | pCas3cRh with the 34 bp crRNA for targeting *hmfI* | This study |

**Table S2:** List of primers used.

| Name | Sequence (5’- 3’) | Template | Application |
| --- | --- | --- | --- |
| HmfF US FWD | gctctagaactagtggatccGCCGGTATCGGCGCG | Purified GO16 gDNA | Generating pKnock-HmfF repair template |
| HmfF US REV | GGACAGCAGCAGCCACCG  GTTGGCCTTACCTCGTCATTATTGTTCT |  |  |
| HmfF DS FWD | ATAATGACGAGGTAAGG  CCAACCGGTGGCTGCTGCTGT |  |  |
| HmfF DS REV | ttgatatcgaattcctgcagCCCCTTGGTCTTGCGCG |  |  |
| pKnock for HmfF FWD | AAGCGCGCAAGACCAAGGGGctgcaggaattcgatatcaagcttatcg | pKnock plasmid |  |
| pKnock for HmfF REV | AACGTCGCGCCGATACCGGCggatccactagttctagagc |  |  |
| PsfG US FWD | gctctagaactagtggatccGCTGGCGCTTCATGTTCTTC | Purified GO16 gDNA | Generating pKnock-PsfG repair template |
| PsfG US REV | TTCAAGTAAAGGAAAACGCAT  TGAAGGAGGATTCATGCAAAGC |  |  |
| PsfG DS FWD | TTCAAGTAAAGGAAAACGCAT  TGAAGGAGGATTCATGCAAAGC |  |  |
| PsfG DS REV | gatatcgaattcctgcagGGCAATATCGGCATCTTCAAAGACC |  |  |
| pKnock for PsfG FWD | GAAGATGCCGATATTGCCctgcaggaattcgatatcaagc | pKnock plasmid |  |
| pKnock for PsfG REV | GAAGAACATGAAGCGCCAGCggatccactagttctagagc |  |  |
| PsfA US FWD | gctctagaactagtggatccCCACTTATGCCCAGGCCTTG | Purified GO16 gDNA | Generating pKnock-PsfA repair template |
| PsfA US REV | ACTTCAGATGGCGCAGCTCC  GAATCCTCCTTCAATCAATGGGTC |  |  |
| PsfA DS FWD | CCATTGATTGAAGGAGGATTC  GGAGCTGCGCCATCTGAAG |  |  |
| PsfA DS REV | cttgatatcgaattcctgcagCCTTCGTGCAATCCGCGATG |  |  |
| pKnock for PsfA FWD | CAAGGCCTGGGCATAAGTGGggatccactagttctagagc | pKnock plasmid |  |
| pKnock for PsfA REV | CAAGGCCTGGGCATAAGTGGggatccactagttctagagc |  |  |
| HmfT US FWD | CGTTTTATTTGATGCCTttaattaaGACGTGCACCTGAGCAAGG | Purified GO16 gDNA | Generating pSEVA237C-HmfT repair template |
| HmfT US REV | TCTGCTCATTGCGTTTTCCTGGTTGCCTCACACAAGATGTGTC |  |  |
| HmfT DS FWD | ACATCTTGTGTGAGGCAACCAGGAAAACGCAATGAGCAGATTG |  |  |
| HmfT DS REV | CAGGAGTCCAAGACTAGTGAGCGTGTCAGGGCAATCAC |  |  |
| pSEVA237C FWD | ACTAGTCTTGGACTCCTG |  |  |
| pSEVA237C REV | TTAATTAAAGGCATCAAATAAAACG | pSEVA237C plasmid |  |
| HmfI US FWD | CGTTTTATTTGATGCCTTTAATTAACATTGGCATCACCCGTACCAAG |  | Generating pSEVA237C-HmfI repair template |
| HmfI US REV | TGAAATCGCTGGGGTAAGTGGAACTCCGATTTTTATATTTATTG | Purified GO16 gDNA |  |
| HmfI DS FWD | AATCGGAGTTCCACTTACCCCAGCGATTTCAACGG |  |  |
| HmfI DS REV | AACAGGAGTCCAAGACTAGTCTGCTCACTTGCAAGCGGTG |  |  |
| pSEVA237C FWD | ACTAGTCTTGGACTCCTG | pSEVA23C plasmid |  |
| pSEVA237C REV | TTAATTAAAGGCATCAAATAAAACG |  |  |
| pBT’T for HmfH FWD | AAGTGGCCCACCCGAGCTGActcgagtctagaggagcatgc |  | *In trans* overexpression of HmfH oxidoreductase of *C. basilensis* HMF14 |
| pBT’T for HmfH REV | CGTTCACGCGGGGTATCCATgaattcggtacctcctgtttcctg |  |  |
| HmfH FWD | gaaacaggaggtaccgaattcATGGATACCCCGCGTGAAC |  |  |
| HmfH REV | catgctcctctagactcgagTCAGCTCGGGTGGGCC |  |  |
| pBT’T for PsfG FWD | CGGTGACCCATTGATTGAAGctcgagtctagaggagcatgc | pBT’T plasmid | *In trans* overexpression of PsfG alcohol dehydrogenase |
| pBT’T for PsfG REV | TTTCCATTCAATCTGCTCATgaattcggtacctcctgtttcctg |  |  |
| PsfG FWD | aaacaggaggtaccgaattcATGAGCAGATTGAATGGAAAAGTCG | Purified GO16 gDNA |  |
| PsfG REV | catgctcctctagactcgagCTTCAATCAATGGGTCACCGAAC |  |  |
| pBT’T for PsfGA FWD | ACGTCGGCACGCTTGGTTGActcgagtctagaggagcatgc | pBT’T plasmid | *In trans* overexpression of PsfG alcohol dehydrogenase  and PsfA aldehyde dehydrogenase |
| pBT’T for PsfGA REV | GTTGCCTCACACAAGATGgaattcggtacctcctgtttcctg |  |  |
| PsfGA FWD |  | Purified GO16 gDNA |  |
| PsfGA REV | catgctcctctagactcgagTCAACCAAGCGTGC |  |  |
| HmfTPsfGA FWD | aaacaggaggtaccgaattcCATCTTGTGTGAGGCAACCATGAC | Purified GO16 gDNA | In trans overexpression of HmfT transporter, PsfG alcohol dehydrogenase  and PsfA aldehyde dehydrogenase |
| HmfTPsfGA REV | catgctcctctagactcgagTCAACCAAGCGTGCCGACG |  |  |
| pBT’T for HmfTPsfGA FWD | ACGTCGGCACGCTTGGTTGActcgagtctagaggagcatgc | pBT’T plasmid |  |
| pBT’T for HmTPsfGA REV | GTTGCCTCACACAAGATGgaattcggtacctcctgtttcctg |  |  |

**Table 3:** gRNA/crRNA sequences in CRISPR knockouts

| **Target** | **Knockout System** | **gRNA/crRNA sequence (5’-3’)** |
| --- | --- | --- |
| *hmfF* | CRISPR/Cas9 | AATGGTCGAGCCAACCACGC |
| *psfG* | CRISPR/Cas9 | GGAATGGGATCGGATCATGG |
| *psfA* | CRISPR/Cas9 | ACCACAACAAATTGTTCGCC |
| *hmfT* | CRISPR/Cas3 | CGGAATCACCACGCTGCCGATCAGGTAGATCACC |
| *hmfI* | CRISPR/Cas3 | GCCAACAGCATGGAGAAGATCACGAAACCGACAC |
| CRISPR/Cas3 plasmids | CRISPR/Cas3 plasmid curing | GCACGATATACAGGATTTTGCCAAAGGGTTCGTG |

**Figure S1:** Growth of GO16 WT on 20 mM HMF. Growth was observed only after increasing the starting OD_600_ to 0.25 from 0.05. Culture conditions were as otherwise described in main text.


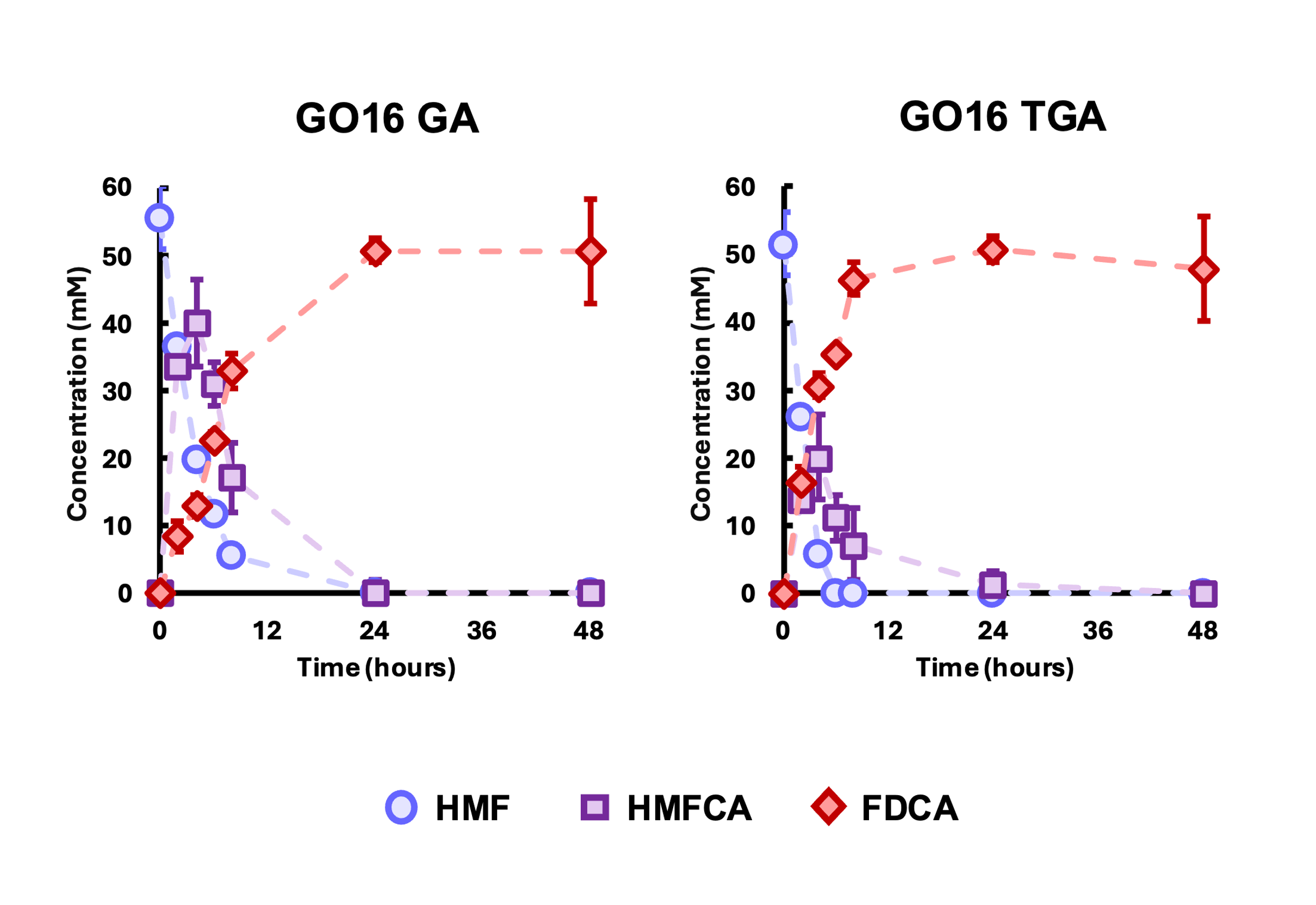


**Figure S2**: Conversion of 50 mM HMF to FDCA by A) GO16 GA and B) GO16 TGA grown on 55 mM glycerol. Error bars represent standard deviation of the mean between biological replicates (*n* = 2).

**
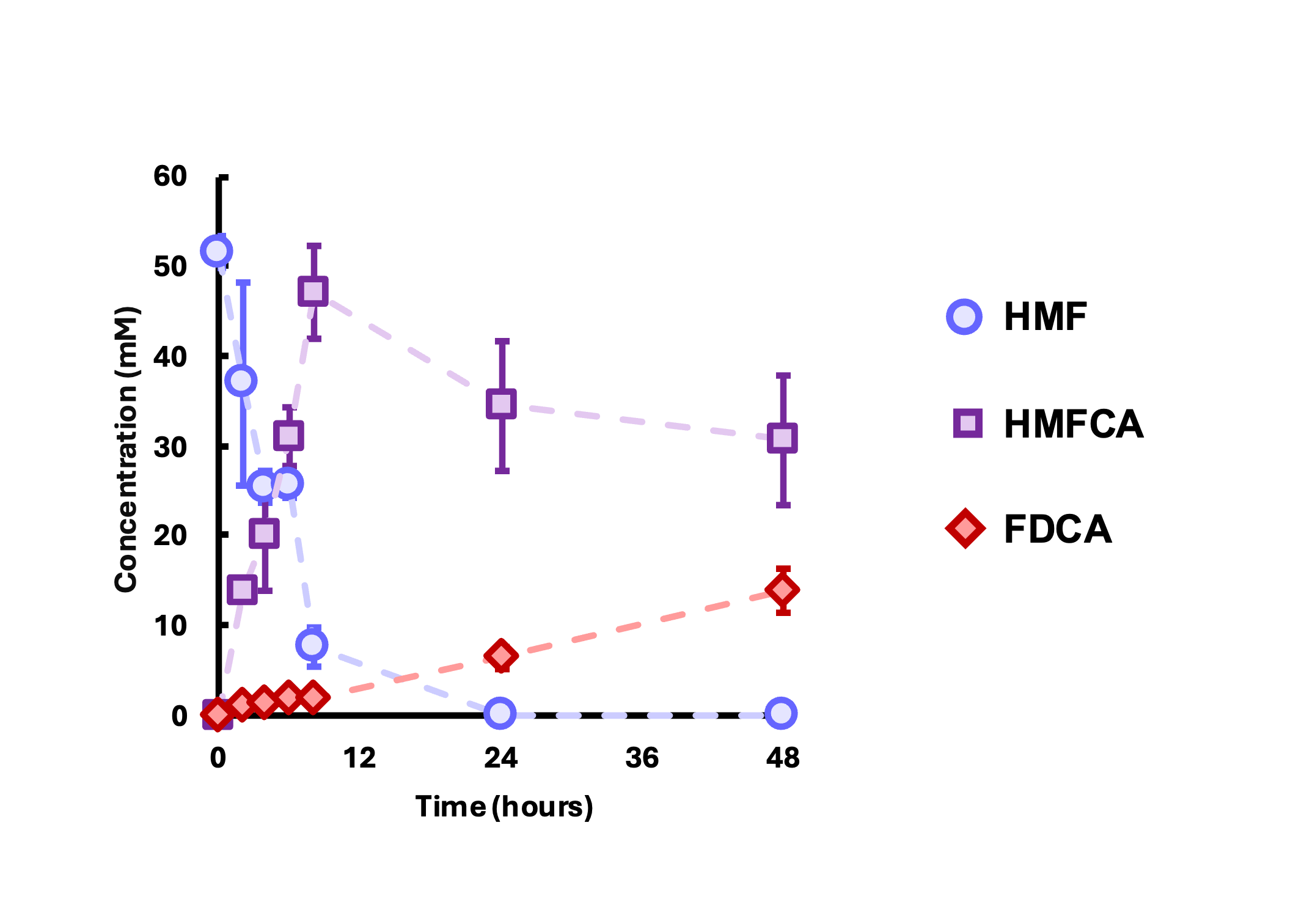
**

**Figure S3**: Conversion of 50 mM HMF to FDCA by GO16 Δ*hmfF* grown on 20 mM TPA but not actively supplemented with it during the biotransformation.. Error bars represent standard deviation of the mean between biological replicates (*n* = 2).

**References**

Csörgő, B., León, L. M., Chau-Ly, I. J., Vasquez-Rifo, A., Berry, J. D., Mahendra, C., Crawford, E. D., Lewis, J. D. & Bondy-Denomy, J. 2020. A compact Cascade–Cas3 system for targeted genome engineering. *Nature Methods,* 17**,** 1183-1190.

Koopman, F., Wierckx, N., De Winde, J. H. & Ruijssenaars, H. J. 2010. Identification and characterization of the furfural and 5-(hydroxymethyl)furfural degradation pathways of <em>Cupriavidus basilensis</em> HMF14. *Proceedings of the National Academy of Sciences,* 107**,** 4919-4924.

Lammens, E.-M., Volke Daniel, C., Schroven, K., Voet, M., Kerremans, A., Lavigne, R. & Hendrix, H. 2023. A SEVA-based, CRISPR-Cas3-assisted genome engineering approach for Pseudomonas with efficient vector curing. *Microbiology Spectrum,* 0**,** e02707-23.

Liu, S., Narancic, T., Davis, C. & O’connor, K. E. 2022. CRISPR-Cas9 Editing of the Synthesis of Biodegradable Polyesters Polyhydroxyalkanaotes (PHA) in Pseudomonas putida KT2440. *In:* MAGNANI, F., MARABELLI, C. & PARADISI, F. (eds.) *Enzyme Engineering: Methods and Protocols.* New York, NY: Springer US.

Silva-Rocha, R., Martínez-García, E., Calles, B., Chavarría, M., Arce-Rodríguez, A., De Las Heras, A., Páez-Espino, A. D., Durante-Rodríguez, G., Kim, J., Nikel, P. I., Platero, R. & De Lorenzo, V. 2013. The Standard European Vector Architecture (SEVA): a coherent platform for the analysis and deployment of complex prokaryotic phenotypes. *Nucleic Acids Research,* 41**,** D666-D675.
